# The leukemogenic fusion gene *MLL-AF9* alters microRNA expression pattern and inhibits monoblastic differentiation via miR-511 repression

**DOI:** 10.1186/s13046-016-0283-5

**Published:** 2016-01-13

**Authors:** Katrin K. Fleischmann, Philipp Pagel, Julia von Frowein, Thomas Magg, Adelbert A. Roscher, Irene Schmid

**Affiliations:** Division of Pediatric Hematology and Oncology, Children’s Research Center, Dr. von Hauner Children’s Hospital, Ludwig-Maximilians-Universität München, Lindwurmstrasse 2a, 80337 Munich, Germany; Lehrstuhl für Genomorientierte Bioinformatik, Technische Universität München, Maximus-von-Imhof-Forum 3, 85354 Freising, Germany; Children’s Research Center, Dr. von Hauner Children’s Hospital, Ludwig-Maximilians-Universität München, Lindwurmstrasse 2a, 80337 Munich, Germany

**Keywords:** Leukemia, AML, MLL, MLL-AF9, microRNA, miR-511, cyclin D1, CCND1, Differentiation, Monocytic differentiation

## Abstract

**Background:**

In this study we explored the role of microRNAs (miRNAs) as mediators of leukemogenic effects of the fusion gene *MLL-AF9*, which results from a frequent chromosomal translocation in infant and monoblastic acute myeloid leukemia (AML).

**Methods:**

We performed a specific and efficient knockdown of endogenous *MLL-AF9* in the human monoblastic AML cell line THP1.

**Results:**

The knockdown associated miRNA expression profile revealed 21 *MLL-AF9* dependently expressed miRNAs. Gene ontology analyses of target genes suggested an impact of these miRNAs on downstream gene regulation via targeting of transcriptional modulators as well as involvement in many functions important for leukemia maintenance as e.g. myeloid differentiation, cell cycle and stem cell maintenance. Furthermore, we identified one of the most intensely repressed miRNAs, miR-511, to raise *CCL2* expression (a chemokine ligand important for immunosurveillance), directly target cyclin D1, inhibit cell cycle progression, increase cellular migration and promote monoblastic differentiation. With these effects, miR-511 may have a therapeutic potential as a pro-differentiation agent as well as in leukemia vaccination approaches.

**Conclusions:**

Our study provides new insights into the understanding of miRNAs as functional mediators of the leukemogenic fusion gene *MLL-AF9* and opens new opportunities to further investigate specific therapeutic options for AML via the miRNA level.

**Electronic supplementary material:**

The online version of this article (doi:10.1186/s13046-016-0283-5) contains supplementary material, which is available to authorized users.

## Background

*MLL-AF9* (alias *KMT2A-MLLT3*) is a fusion gene resulting from the chromosomal translocation t(9;11)(p22;q23). It is sufficient to initiate acute leukemia in murine models [1, 2] and is the most frequent fusion gene in infant acute myeloid leukemia (AML) and especially associated with monoblastic AML (FAB-classification M5) [3, 4]. For this type of leukemia with a persisting poor prognosis, new targeted therapies are needed.

MLL and AF9 wildtype proteins lead to transcriptional initiation (MLL) and elongation (AF9) of target genes [5, 6], while the fusion protein MLL-AF9 is believed to combine these properties, leading to increased activation of target genes [7]. Novel therapeutic strategies which aim to directly intervene with MLL-AF9 functions are under investigation but struggle with the complexity to target the involved protein-protein and protein-DNA interactions and with the obstacle to avoid targeting of the important wildtype protein functions [5, 7].

MicroRNAs (miRNAs) are important cellular posttranscriptional gene regulators and are involved in presumably all physiological and pathophysiological cellular processes [8], including oncological state, progression and clinical outcome [9–11].

Although technical challenges – predominantly the specific, efficient and safe delivery of miRNA modulators to the tumor site – remain to be overcome, miRNA therapeutics are believed to hold huge promise for cancer treatment [12]. In this context, a comprehensive understanding of the role of miRNAs within the complex regulatory networks in malignant cells is a prerequisite for the development of combinatorial therapeutic strategies employing miRNA modulators [12].

As fusion gene breakpoints vary between individual patients, siRNA-targeting of oncogenic fusion genes themselves would have to be tested and optimized in each case and thus does not represent an opportune therapeutic option. Finding common, relevant downstream miRNA targets of MLL-AF9 instead might yield new alternative therapeutic routes. This approach has the benefit that oncogenic transcription factors or other protein classes which are currently non-druggable by e.g. small molecule drugs might be targeted via miRNA modulators. Another advantage of miRNA therapeutics is the fact that not only single gene transcripts can be targeted (e.g. via target site blockage) but also whole arrays of mRNAs, which may cooperate to create an oncogenic phenotype (e.g. via miRNA mimics or inhibitors).

In order to identify new alternative drug targets for *MLL-AF9* positive AML, we thus aimed to explore the role and function of miRNAs as mediators of leukemogenic effects of the fusion gene *MLL-AF9*. To this end, a differential miRNA expression profile was generated after knockdown of endogenous *MLL-AF9* in the monoblastic AML cell line THP1 and revealed 21 miRNAs predicted to be involved in leukemogenic functions. Additionally, we provide evidence for a relevant role of miR-511 in the context of monoblastic differentiation. To our knowledge, this is the first experimental study that comprehensively analyzes the effects of an *MLL* fusion gene on miRNA expression.

## Methods

### Cells, cell cultivation, small interfering RNA and transfection

Cultivation and siRNA treatment of THP1 cells (DSMZ GmbH, Braunschweig, Germany) was performed as previously reported [7]. Briefly, the cell line was authenticated via MLL-AF9 breakpoint sequencing, was routinely checked for mycoplasma contamination and transfected with 50 nM Silencer Select siRNAs (Life Technologies, Carlsbad, CA, USA) and Dreamfect (OZ Biosciences, Marseille, France) leading to transfection efficiencies and survival rates of 93 % each. Experimental incubations lasted eight days with repeated transfections on day 0, 3, and 6. Prior to each transfection event, cell densities were determined and cells were reseeded at 5 × 10^4^ cells per ml [7].

THP1 miRNA mimic transfections were performed as described for siRNA transfections but with 30 nM Ambion Pre-miR miRNA Precursors (hsa-miR-511-5p AM10237, neg Control #1 AM17110, neg Control #2 AM17111) and experimental incubations up to day nine again with repeated transfections on day 0, 3, and 6.

Human CD14+ monocytes (C-12909, PromoCell GmbH, Heidelberg, Germany) were cultivated for 48 h in mononuclear cell medium (PromoCell) prior to immunostaining and RNA isolation. Human CD34+ progenitor cells (C-12921, PromoCell) were directly taken to RNA isolation.

### RNA isolation and reverse transcription quantitative PCR assays and arrays

Total RNA was extracted with miRNeasy Mini Kit (Qiagen, Hilden, Germany) according to the supplier’s protocol. For miRNA profiling, RNA of five independent experiments was pooled for each of the four distinct siRNA treatments to reduce inter-experimental variation.

According to the supplier’s protocol, quantitative miRNA profiles of 664 distinct miRNAs were generated employing 350 ng total RNA per array card, preamplification step and TaqMan Array Human MicroRNA Cards v2.0 (also known as TaqMan Low Density Arrays (LDAs)) on an ABI PRISM 7900HT machine with SDS 2.3 software (all Life Technologies). LDA raw data were imported into the statistical language R version 2.11.1 [13]. As it is sometimes difficult to define reliable housekeeper in the case of miRNAs, it has been shown that normalizing to the overall mean instead may outperform the reference approach [14]. Thus, a cyclic LOESS normalization was applied to the data (separately for the A and B plates). Analysis of differential expression between *MLL-AF9* knockdown and control treatments was carried out in the statistical language R with the limma package [15]. Adjustment for multiple testing was done using the method by Benjamini and Hochberg [16]. Log_2_FC, *p*-value of the moderated *t*-test and *p*-value after correcting for multiple testing were generated. MiRNAs which showed low expression, i.e. high threshold cycle (C_T_ values above 34.5) in both treatment groups were excluded from the analysis. MiRNAs with a *p*-value below 0.05 were regarded as differentially expressed. MiRNAs which were either turned “on” or “off” in both *MLL-AF9* knockdown versus both non-targeting control samples (“undetermined” versus CTs below 34.5) were added to the miRNA candidate list manually. For these, no ratio can be calculated but a lower limit of differential expression is reported as previously described [17]. Of the 664 distinct miRNAs detectable by this LDA, 72 are measured in duplicate. MiRNAs were removed from the candidate list if their replicate did not show concordant regulation. MiRNA profiling data have been deposited in and approved by NCBI’s Gene Expression Omnibus (GEO) [18] and are available under the accession number GSE70525.

For LDA validation, pooled RNA samples from two additional, independent replicate experiments was subjected to quantitative real time PCR via single TaqMan® miRNA assays after megaplex reverse transcription with TaqMan MicroRNA Reverse Transcription Kit (Life Technologies) and preamplification according to the manufacturers’ instructions. For this purpose, the following miRNA assays were used (Life technologies, assay IDs given in parentheses): hsa-miR-214 (002306), −219-5p (000522), −369-3p (000557), −432 (001026), −511-5p (001111), −539 (001286), −576-5p (002350), −582-3p (002399), −589 (002409), −758 (001990) and −760 (002328). RNU6B (001093), RNU44 (001094) and RNU48 (001006) assays were tested as internal references. RNU48 showed the least intra- and inter-experimental variance and thus was used as internal reference. Spearman’s rank correlation coefficient including *p*-value was calculated with the statistical language R version 2.11.1 [13]. MiR-511 quantification in further experiments was performed via TaqMan miRNA assays after concurrent (2-plex) reverse transcription for hsa-miR-511 and the internal reference RNU48 from 200 ng total RNA.

For gene expression quantification, reverse transcription quantitative PCR (RT-qPCR) was carried out in triplicates with 600 ng total RNA input, QuantiTect Reverse Transcription Kit (Qiagen) and iQ-SYBR Green Supermix (Biorad, Hercules, CA, USA) on a StepOnePlus instrument (Life Technologies) according to the supplier’s protocol. *RPL13A* and *UBC*, showing stable expression in bone marrow [19], were used as reference genes. Data were analyzed with StepOne software v 2.1 (Life Technologies) and the ∆∆C_T_ method. Primers were designed utilizing Clone Manager Suite 7 (Sci-Ed Software, Cary, NC, USA) and validated for efficiency via cDNA dilution series and for specificity via PCR-product analysis on agarose gels and melt curve analysis. Primer sequences are provided in Additional file 1: Table S1.

### Gene expression profiling analysis

Human Whole Genome Microarrays 4x44K v2 (Agilent Technologies, Santa Clara, CA, USA) were commissioned to and performed at IMGM Laboratories (Martinsried, Germany). For hybridization, 100 ng RNA of two independent THP1 experiments (harvested two days after transfection with miR-511 or negative control #1 mimics) were utilized. RNA concentration and purity (abs 260/280 nm) were analyzed using a Nanodrop ND-1000 Spectrophotometer (Nanodrop Technologies, Wilmington, DE, USA). RNA integrity was determined with an RNA 6000 Nano LabChip Kit on a 2100 Bioanalyzer (Agilent Technologies). A260/A280 was above 2.0 and RNA integrity number was above 9.5. Between-array normalization was performed with the quantile method.

Analysis of differential expression was carried out in R [13] with the limma package [15, 20]. Adjustment for multiple testing was done using the method by Benjamini and Hochberg [16].

Probes were considered as differentially expressed at a *p*-value of the moderated *t*-test below 0.05 and significant alteration over all identical replicate probes (*t*-test, *p* < 0.05). Gene expression profiling data have been deposited in and approved by NCBI’s Gene Expression Omnibus (GEO) [18] and are available under the accession number GSE70490.

### Functional bioinformatics

MiRTarBase v4.5 [21] was used to extract known, validated miRNA targets of the set of 21 MLL-AF9 dependently expressed miRNAs. Likewise, TargetScan release 6.2 [22] was employed to predict putative targets of this set of miRNAs.

Functional annotation analysis (Database for Annotation, Visualization and Integrated Discovery version 6.7, DAVID) [23, 24] was performed with validated miRNA targets from miRTarBase (excluding those with weak experimental evidence) as well as with putative miRNA targets predicted via TargetScan.

MiRNA targets with strong experimental support in miRTarBase underlie individual experimental validation for each miRNA-target gene interaction. As this requires considerable experimental effort, miRTarBase targets will not represent a random selection of genes but have a bias for genes with known, important biological and pathophysiological functions. It is thus not surprising to find an enrichment for disease- and cancer-related gene ontology terms in any subset of genes listed in miRTarBase (especially for those with strong experimental support). Due to this bias, we did not interpret these gene ontology results in respect to their magnitude of enrichment but rather used the resulting ontology terms as a description of the biological roles these miRNA target genes are known to play. To integrate the cellular background, we regarded only those miRNA targets for these analyses, which were expressed in our THP1 gene expression profile [7].

To limit the bias of speculative interference of TargetScan predictions, we employed a stringent context + score cutoff of at least −0.4 or +0.4 for the sum of all miRNA-target gene interactions to regard a gene as predicted target of one or more of our set of miRNAs. The context + score is given by TargetScan and represents the likelihood of a miRNA-target interaction ( MTI). To compile the sum of context + scores, MTIs of down-regulated miRNAs (in *MLL-AF9* knockdown) retained negative context + score values (original TargetScan values), while context + score values of MTIs of up-regulated miRNAs were modified to positive values. As recommended, DAVID gene ontology enrichment was regarded as significant if fold enrichment was ≥ 1.5 and *p*-value < 0.1.

Functional disease ontology (FunDO) [25] was performed with differentially expressed genes in miR-511 mimic treated THP1 cells to extract genes involved in cancer and leukemia.

### Vector construction and luciferase reporter assay

*FGFR3* and *PDGFA* full length 3′UTRs were amplified from THP1 genomic DNA and THP1 cDNA respectively, *CCND1* full length 3′UTR was amplified and subcloned from an pcDNA3.1LacZ-*CCND1*-3′UTR vector [26] (generously provided by Katherine L.B. Borden, Université de Montréal, Canada). Overlapping PCR was used to introduce *CCND1*-3′UTR deletions of miR-511 binding site seed regions predicted via the algorithms DianaMiRExtra, TargetScan, DIANA-microT-CDS (v5.0) and miRDB. All 3′UTRs were cloned into siCHECK2 vector (Promega, Madison, Wisconsin, USA) and fully sequenced (Eurofins Genomics, Ebersberg, Germany) to confirm correct 3′UTR sequences.

Luciferase reporter assays were performed after transfection of HEK-293 cells with vector (100 ng/well) in combination with either miR-511 or negative control mimic (20 pmol/well). Experiments were carried out in 24-well format with 7.5 × 10^4^ cells seeded in 1 ml medium (DMEM high glucose, 10 % FCS) 24 h prior to transfection with 2 μl Lipofectamine 2000 (Life Technologies). Cells were harvested 24 h after transfection and Dual-Luciferase® Reporter Assays (Promega, Fitchburg, WI, USA) were performed according to supplier’s protocol on a Fluostar Optima (BMG Labtech GmbH, Ortenberg, Germany).

### Biological assays and flow cytometry analyses

Cell numbers and proliferation rates were determined via MoxiZ automated cell counter (Orflo, Ketchum, ID 83340, USA).

Cell cycle analyses were performed in combination with anti-CCND1 staining via flow cytometry. Cells were fixed in cold methanol at −20 °C, blocked with human IgG (I 2511, Sigma-Aldrich, St. Louis, MO, USA) and stained with FITC Mouse Anti-Human Cyclin D1 Antibody Set (#554109, BD Biosciences, Franklin Lakes, NJ, USA,) and propidium-iodide (0.1 % Triton X-100, 0.2 mg/ml RNase A, 20 μg/ml propidium-iodide in PBS) and analyzed on a BD FACSCanto (BD Biosciences). Cell doublets and aggregates were removed by gating and the proportion of cells in G0/G1, S and G2 phase were quantified with Watson Pragmatic model in FlowJo version 9.7.2 (FlowJo LLC, Ashland, Oregon, USA).

Colony forming capacity was analyzed by plating 200 THP1 cells in 200 μl (10000 cells/ml) 0.5 % methyl cellulose (64630, Sigma-Aldrich) / RPMI1640 (10 % FCS) in 24-well plates. Colonies were counted in microscopic images taken after 10 and 14 days by Cellscreen system (Innovatis, Bielefeld, Germany) on an Olympus IX 50 microscope (Olympus, Tokyo, Japan).

The effect on monocytic cell differentiation was analyzed via flow cytometry and surface staining with AF488 mouse anti-human CD11b, PE mouse anti-human CD38 and isotype control antibodies (BD561687, BD557702, BD560981, BD555749, BD Biosciences) after blocking Fc receptors with FcR blocking reagent (BD564220, BD Biosciences). To establish CD11b and CD38 flow cytometric measurements and for determining effects of differentiation on miR-511 expression, THP1 cells were treated with 0.25 – 5.0 ng/ml PMA (P1585, Sigma-Aldrich) and monocyte / macrophage differentiation was confirmed via cell adherence, morphological changes (cell protrusions, fusiform cells) and growth arrest.

Cellular migration was analyzed in transwell assays (5 μm pore size, #3421, Corning, NY, USA) with 1 × 10^5^ THP1 cells in RPMI1640 / 10 % FCS in upper chamber and RPMI1640 with 10 % FCS and 0.1 mM ascorbic acid in lower chamber. Cells in lower chamber were counted by MoxiZ cell counter (Orflo) and in microscopic images taken after 20 h by Cellscreen System (Innovatis). To test for significant differences between treatments, student’s *t*-tests were performed.

## Results

### MLL-AF9 depletion leads to changes in miRNA expression

To explore if expression of the leukemogenic fusion gene *MLL-AF9 (KMT2A-MLLT3)* leads to changes in miRNA expression, a specific and efficient knockdown of endogenous MLL-AF9 was performed in the human monoblastic AML cell line THP1 as previously described [7]. To exclude siRNA off-target effects, these experiments employed two distinct *MLL-AF9* specific as well as two distinct control siRNAs. Experimental incubations lasted eight days with repeated transfections on day 0, 3, and 6. Knockdown reduced *MLL-AF9* to 22.3 ± 6 % residual expression on transcript level and to 8 ± 4 % on protein level on day 8 of experiments. *MLL* and *AF9* wildtype transcript levels were not significantly altered. Additionally, mRNA reduction of *HOXA9* (a known direct target of MLL-AF9) to 56.9 ± 8 % residual expression confirmed the knockdown on functional protein level [7].

In this setting, we previously reported a specific gene expression signature shaped by MLL-AF9 together with affected downstream processes and new potential therapeutic targets [7].

The specificity of the knockdown experiments was confirmed by (1) the presence of important marker genes and (2) the detection of significant enrichments of direct MLL-AF9 targets identified in a mouse model and (3) of genes downstream of MLL-AF9 identified in transduced primary human cells [7]. Here, we analyzed the corresponding miRNA expression profile (on experimental day 8) with the goal to detect additional MLL-AF9 dependent effects via this important posttranscriptional regulatory mechanism. MiRNA profiling data have been deposited in and approved by NCBI’s Gene Expression Omnibus (GEO) [18] and are available under the accession number GSE70525.

**Fig. 1:**
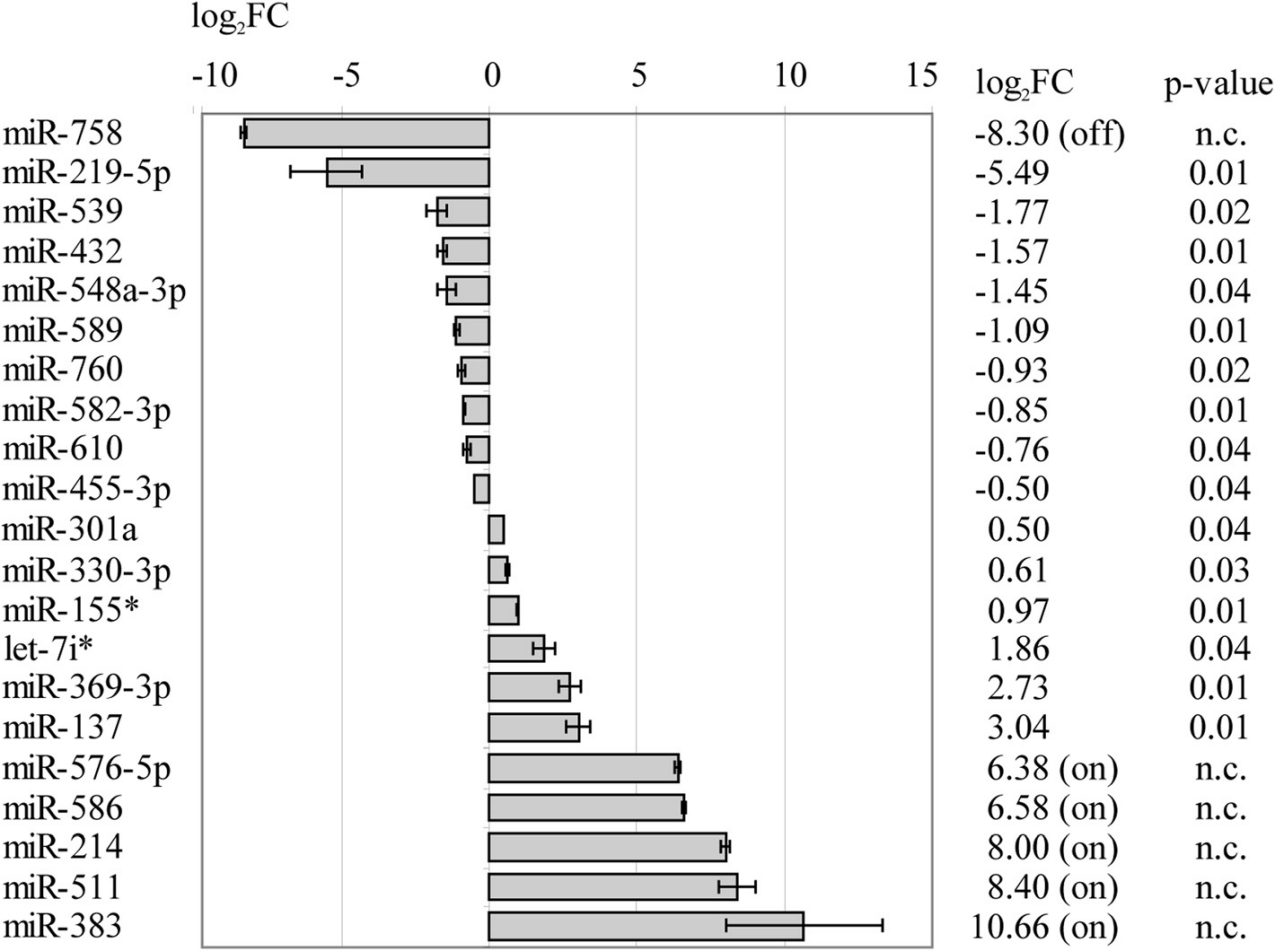
Differentially expressed miRNAs after *MLL-AF9* knockdown in THP1 cells. TaqMan low density array analysis was utilized for quantification of differential expression which is shown relative to non-targeting control siRNA treatments. Bars indicate standard error of the mean. Columns on the right show log_2_ fold change (log_2_FC) and *p*-value of the corresponding miRNA. For miRNAs which were turned “on” or “off”, i.e. not detectable in either both control or both *MLL-AF9* knockdown treatments, a lower limit of differential expression is reported as described in methods. Here no *p*-value can be calculated. n.c., not calculable

We detected 21 miRNAs as differentially expressed upon *MLL-AF9* depletion (Fig. 1). For this analysis, miRNA expression was screened via TaqMan low density arrays (LDAs) and revealed above background detection of 53 % of all 664 analyzed miRNAs. Among those, 15 reached significant differential expression (*p* < 0.05) and additional 6 miRNAs were either turned “on” or “off” in both *MLL-AF9* knockdown versus both control treatments. For these, no exact ratio can be determined but - as previously described [17] - a lower limit of differential expression was determined (Fig. 1). A significant correlation between LDA data and single-assay RT-qPCR data (performed on pooled samples from two additional independent experiments) for 11 of the differentially expressed miRNAs (*p* 0.03, Spearman’s *Rho* 0.66) supported the screening results (Additional file 2: Figure S1).

### Functional role of miRNAs as conferred from validated and predicted miRNA targets

Experimentally confirmed targets (extracted from miRTarBase v4.5 [21]) of MLL-AF9 dependently expressed miRNAs, which were expressed in THP1 cells, were used to identify functions and pathways impaired by *MLL-AF9* fusion gene via miRNA expression. MiRTarBase held 328 validated miRNA-target interactions (MTIs) for 14 out of our set of 21 MLL-AF9 dependently expressed miRNAs. However, we excluded 265 of these, which were marked for weak experimental support, leaving 63 MTIs with strong experimental support. To integrate the cellular background, we regarded only those miRNA targets for these analyses, which were expressed in our THP1 gene expression profile [7]. This resulted in 56 remaining MTIs and contained 44 genes as validated targets for 10 out of our set of 21 differentially expressed miRNAs. In order to interpret the functional roles which these miRNAs may exert via these known 44 target genes, we performed a gene ontology analysis with DAVID software [23, 24] (Additional file 1).

To structure the results, we manually selected biological relevant terms in the context of myeloid leukemia, excluded redundant terms and assorted the terms to higher-order terms (Additional file 2: Figure S2). The results especially indicated a role of the targets – and thus inferred a role of the miRNAs – in proliferation and apoptosis, in functions related to monocyte/macrophage differentiation as well as in transcriptional regulation and cancer related functions. Further analysis revealed that five of the miRNAs (miR-137, −214-3p, −301a-3p, −330-3p and −383-5p) are known to target genes affecting all of these four categories (Additional file 2: Figure S3), implying their relevance for leukemia as well as the possibility to therapeutically target gene regulation via these miRNAs.

Confirmed targets with strong experimental support (as listed in miRTarBase) were known only for a subset of the MLL-AF9 regulated miRNAs (10 out of 21). As these miRNA-target studies are not all-encompassing, these known targets will additionally represent only a fraction of the actual effective and relevant miRNA targets. Thus, the performed analysis of known, experimentally confirmed and THP1-expressed direct targets can only give partial insights into the roles of these miRNAs. To overcome these limitations, we additionally analyzed the roles of predicted targets for all of the 21 differentially regulated miRNAs.

**Fig. 2:**
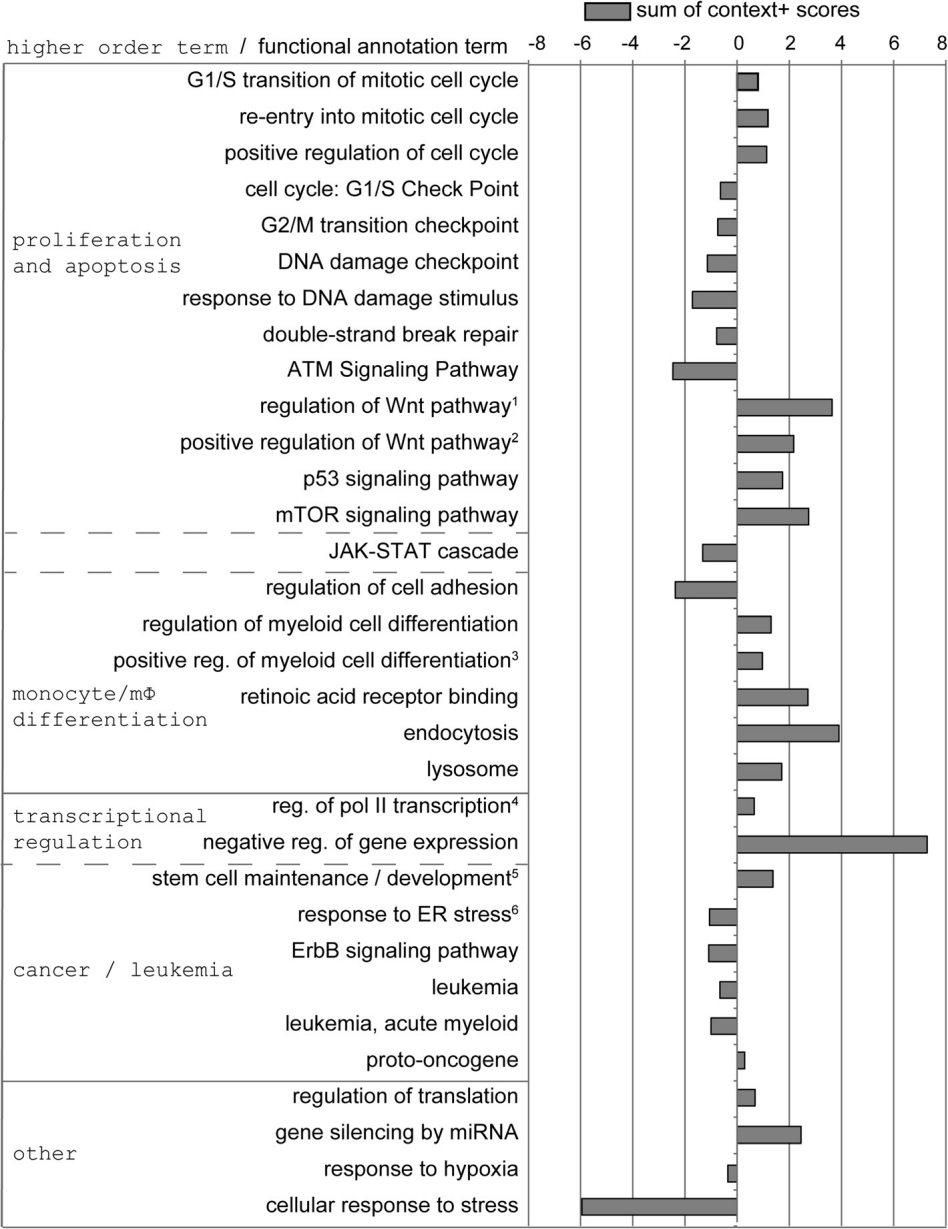
MiRNA-target gene prediction associated functional annotation terms identified via DAVID. Gene ontology analysis was performed for all THP1-expressed, targets predicted via TargetScan with a minimum context + score of ±0.4 of *MLL-AF9* knockdown associated miRNAs. Annotation terms were manually assorted to five higher-order terms according to the major role of the process in the biological setting under investigation. Annotation terms assigned to two higher-order terms are placed between these two and separated by dotted division lines. Some annotations were abbreviated as indicated by superscript numbers: ^1^regulation of Wnt receptor signaling pathway, ^2^positive regulation of Wnt receptor signaling pathway, ^3^positive regulation of myeloid cell differentiation, ^4^regulation of transcription from RNA polymerase II promoter, ^5^stem cell maintenance / stem cell development (two synonymous terms), ^6^response to endoplasmic reticulum stress. Columns represent the sum of context + scores for all MTIs of this term, where MTIs of down-regulated miRNAs (in *MLL-AF9* knockdown) retained negative context + score values, context + score values of MTIs of up-regulated miRNAs received positive values. Thus negative values of this column indicate strong predicted targeting by down-regulated miRNAs and positive values indicate strong predicted targeting by upregulated miRNAs, while targets which were similarly likely targeted by up- and down-regulated miRNAs do not reach the sum of context + score cutoff and are not included in the predicted target gene list

Among numerous miRNA target prediction algorithms, TargetScan [22] is the most popular and widely used and has recently been evaluated to yield one of the best predictive performances [27]. TargetScan predicted targets for 19 out of our set of 21 MLL-AF9 dependently expressed miRNAs. To limit the bias of speculative interference, we employed a stringent context + score cutoff to regard a gene as predicted target of one or more of the set of 19 miRNAs (see methods for details). Again, only those targets which were expressed in THP1 cells [7] were included in the analyses. This approach yielded 5500 predicted MTIs concerning 1827 predicted target genes (most genes were predicted targets of more than one miRNA) for which gene ontology analyses were performed via DAVID software (Additional file 3). The results, filtered for biologically relevant and non-redundant terms are presented in Fig. 2.

Remarkably, ontology terms concerning cell cycle progression (G1/S transition of mitotic cell cycle, re-entry into mitotic cell cycle, positive regulation of cell cycle) were predominantly predicted as targets of miRNAs up-regulated in the context of MLL-AF9 depletion, while terms related to cell cycle checkpoints (cell cycle: G1/S checkpoint, G2/M transition checkpoint, DNA damage checkpoint) were predominantly targeted by down-regulated miRNAs (see sum of context + score of terms in Fig. 2), implying a cell cycle promoting and checkpoint decreasing role of MLL-AF9 via miRNAs.

Stem cell maintenance/development and cellular response to stress were predominantly predicted to be targeted by MLL-AF9 dependently down-regulated and up-regulated miRNAs respectively, suggesting a stem cell supporting and cellular stress response suppressive role of MLL-AF9 via miRNAs.

To compare the gene ontology results of MLL-AF9 dependently regulated genes [7] with those of known and predicted targets of MLL-AF9 dependently regulated miRNAs, we compiled a table with related gene ontology terms that were present in at least two of the three data sets (Table 1). This overview illustrates that genes directly involved in the cell cycle, Wnt signaling, adhesion, myeloid differentiation as well as in the cellular response to stress are preferentially present in the miRNA target gene data sets and thus these processes may be preferentially influenced by MLL-AF9 via miRNA expression.

**Table 1:** Comparison between gene ontology results of MLL-AF9 dependently expressed genes and miRNA target genes

	Gene expression	Known miRNA targets	Predicted miRNA targets
DNA replication	×	Ø	×
apoptosis	×	×	Ø
**cell cycle**	Ø	×	×
JAK-STAT signaling	×	Ø	×
**Wnt signaling**	Ø	×	×
cytokine	×	×	Ø
endocytosis	×	Ø	×
**adhesion**	Ø	×	×
lysosome	×	Ø	×
**myeloid differentiation**	Ø^a^	×	×
hemopoiesis	×	×	Ø
transcription factor	×	×	×
reg of pol II transcription	×	×	×
cell size	×	×	Ø
**cellular response to stress**	Ø	×	×

Compared were the gene ontology results obtained via DAVID software between MLL-AF9 dependently expressed genes [7] and known (miRTarBase) as well as predicted (TargetScan) target genes of MLL-AF9 dependently expressed miRNAs. × present in the data set, Ø not present in the data set

^a^refers to gene ontology terms directly concerning myeloid differentiation (e.g. “regulation of myeloid cell differentiation”), whereas terms indirectly related to myeloid differentiation (e.g. “phagocytosis”) were also present within the gene expression profile. Terms in bold letters were preferentially present in the miRNA target gene data sets

### Gene regulatory effects of miR-511 expression in THP1 monoblasts

While the preceding ontology analyses may suggest certain functional roles of the set of MLL-AF9 differentially regulated miRNAs in the context of leukemia, one has to take into account that these functions are always context dependent and will have to be experimentally validated. To this end, we decided to further experimentally investigate the functional role of miR-511, one of the most potently activated miRNAs after MLL-AF9 depletion. Concerning known targets, so far only TRIB2, TLR4 and CD80 have been validated for miR-511 in other cellular contexts [28, 29].

To identify targets and effects of miR-511 in monoblasts, we performed miR-511 up-regulation in THP1 cells by transfecting miRNA mimics. The subsequently analyzed gene expression profiles via whole genome arrays revealed 653 probes representing 627 genes to be differentially expressed between miR-511 and negative control mimic treatments (profiling data have been deposited in NCBI’s Gene Expression Omnibus (GEO) [18] and are available under the accession number GSE70490). Of these, 45 % were down-regulated. The previously described miR-511 target genes were either not expressed above background (TRIB2 and CD80) or not differentially regulated (TLR4) on transcript level in this setting.

**Fig. 3:**
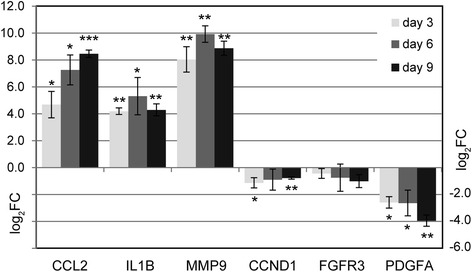
MiR-511 dependently expressed gene transcripts. Subsequent to introduction of miR-511 mimics in THP1 cells, differential expression of a subset of potentially important transcripts identified in the gene expression screen was verified in three additional independent experiments via RT-qPCR on experimental days 3, 6 and 9. Columns indicate log2 fold change (log_2_FC) between miR-511 and negative control #1 mimic treatments. Bars indicate standard error. * *p* < 0.05, ** *p* < 0.005, *** *p* < 0.0005

Based on links to leukemia or cancer via functional disease ontology (FunDO [25], Additional file 4) or literature research, we selected 15 out of 627 differentially expressed genes after miR-511 up-regulation in THP1 cells for confirmation via RT-qPCR (Additional file 2: Table S1). Of these, five genes were confirmed to be significantly differentially regulated after miR-511 transfection in three additional independent replicate experiments (Fig. 3). These were the up-regulated genes chemokine (C-C motif) ligand 2 (*CCL2*), matrix metallopeptidase 9 (*MMP9*) and interleukin 1 beta (*IL1B*) as well as the down-regulated genes cyclin D1 (*CCND1*) and platelet-derived growth factor alpha polypeptide (*PDGFA*). For fibroblast growth factor receptor 3 (*FGFR3*) a trend towards down-regulation was observed (Fig. 3).

### MiR-511 directly regulates cyclin D1

**Fig. 4:**
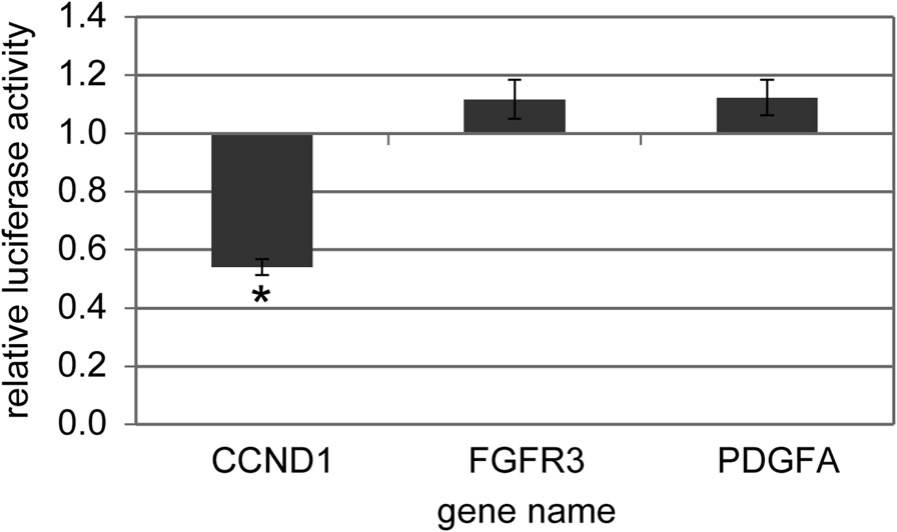
Effect of miR-511 mimic on 3′UTRs of selected genes in a dual luciferase reporter assay. Columns represent mean over five independent experiment, bars indicate standard error of the mean. * *p* < 0.05 in all replicate experiments

For the three genes which were down-regulated on transcriptional level after miR-511 treatment (*CCND1*, *FGFR3* and *PDGFA*), luciferase reporter assays were performed to test for direct miR-511 targeting. Full-length 3′UTRs of these transcripts were cloned into siCHECK2 vector. *FGFR3*- and *PDGFA*-3′UTRs were not affected by miR-511 in dual luciferase assays while *CCND1*-3′UTR was targeted by miR-511 as indicated by significantly reduced *Renilla* luciferase signals (Fig. 4).

**Fig. 5:**
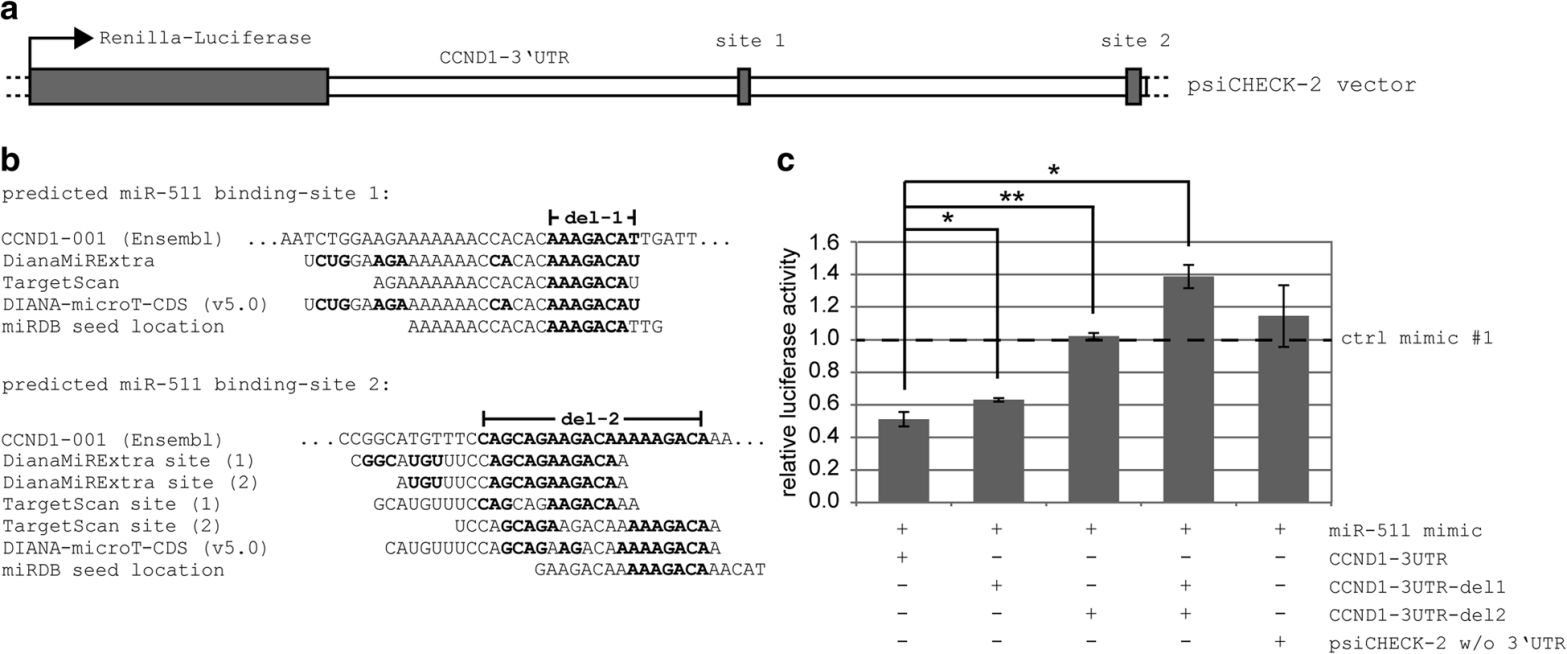
Effect of miR-511 on *CCND1*-3′UTR with and without deletion of miR-511 binding site seed regions. **a** Structural map of siCHECK2 vector: Wildtype full length *CCND1*-3′UTR consists of 3192 nucleotides and was cloned into the multiple cloning region located downstream of the *Renilla* luciferase translational stop codon of siCHECK-2 vector. Predicted miR-511 binding sites are indicated. **b** Sequence of miR-511 binding sites as predicted by the indicated algorithms. *CCND1*-3′UTR nucleotides complementary to miR-511 are indicated in bold. Positions 2738–2745 of *CCND1*-001 Ensemble transcript variant were deleted for binding site 1 seed region (del-1) and positions 4251–4270 for binding site 2 seed region (del-2). **c** Effect of miR-511 mimic on *CCND1*-3′UTR with and without seed region deletions in dual luciferase reporter assay. Columns represent mean over three independent experiments, bars indicate standard deviation; * p ≤ 0.05; ** p ≤ 0.005; w/o, without

To further validate the direct interaction and to determine which binding sites for miR-511 are relevant for *CCND1* regulation, seed sequences of predicted binding sites were deleted (Fig. 5a, b) and again dual luciferase assays were performed. Here, binding site 1 did contribute significantly to *CCND1*-downregulation although only to a small extent, while binding site 2 deletion was able to completely abrogate regulation through miR-511 (Fig. 5c). Parallel deletion of both binding sites even led to an up-regulation of protein expression (Fig. 5c).

This phenomenon could be theoretically due to an off-target binding of the employed negative control mimic to the *Renilla* luciferase- or the subsequent 3′UTR-sequence. However, using negative control mimic #2 and no mimic as further controls did yield similar results as with negative control mimic #1 and thus disagree with this hypothesis. Another possible explanation would be an off-target binding of miR-511 to the housekeeping firefly luciferase sequence, however, sequence comparisons do not support this possibility and, more importantly, the luciferase expression from the vector without 3′UTR is not affected by miR-511 treatment (compared to negative control mimics or no mimic). We thus currently cannot define the cause of the up-regulatory effect of miR-511 in luciferase assays when both binding sites were deleted from *CCND1*-3′UTR. However, the employed controls indicated that this observation was not due to a miRNA-mimic off-target binding and that the effect is dependent on the presence of *CCND1*-3′UTR.

### MiR-511 reduces cyclin D1 protein level and percentage of cells in S-phase in THP1

**Fig. 6:**
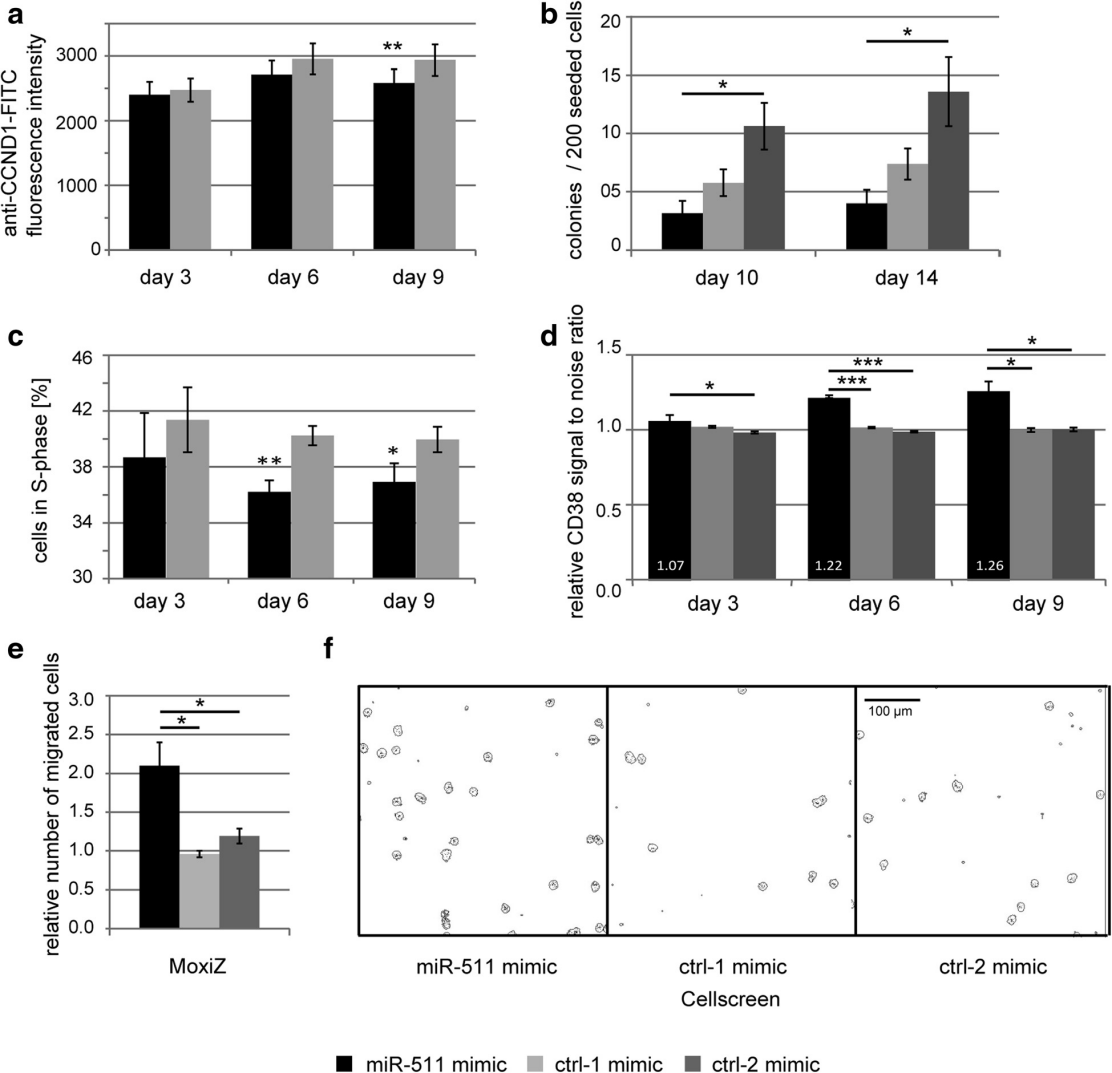
Cellular effects of miR-511 mimic treatment in THP1 cells. **a** Effect of miR-511 mimic treatment on cyclin D1 protein expression of THP1 cells measured by flow cytometry (median fluorescence intensity). **b** Mean colony number formed by THP1 cells 10 and 14 days after having been transfected three times over a time frame of 10 days with miRNA mimics. **c** Effect of miR-511 mimic treatment on proportion of THP1 cells in S-phase. **d** Effect of miR-511 mimic on surface expression of monocytic marker CD38. **e** Effect of miR-511 mimic treatment on migratory capability of THP1. On day 8 after miRNA mimic transfection, cells were left migrating through 5 μm transwells for 20 h. Number of migrated cells was counted via MoxiZ cell counter and Cellscreen microscopy with equal results. **f** Representative pictures of Cellscreen microscopy of migrated THP1 cells are shown. Columns show mean over four (**a**, **c**) or three (**b**, **d**, **e**) independent experiments, bars indicate standard error of the mean. Black: miR-511 mimic treatment, light grey: negative control #1 mimic treatment (ctrl-1 mimic), dark grey: negative control #2 mimic treatment (ctrl-2 mimic). * *p* < 0.05; ** *p* < 0.005

In the next step we examined the effect of miR-511 on the protein expression of cyclin D1 (*CCND1*) via flow cytometric analysis of cyclin D1 stained THP1 cells transfected either with miR-511 or control #1 mimic. Time course experiments showed a trend towards reduced cyclin D1 protein expression on day 6 and significant reduction on day 9 after miR-511 mimic treatment (Fig. 6a).

Due to the experimental design with reseeding of cells prior to mimic transfections, cell proliferation could only be assessed over a period of 3 days in a row, within which no significant difference between miR-511 mimic and control mimic treatments was observed. Concerning an effect of miR-511 on the colony formation capacity of THP1 monoblasts, we observed a significant reduction of colony numbers after miR-511 mimic treatment when compared to negative control #2 mimic (p 0.02 for day 10 and 14), but only a trend towards reduced colony numbers when compared to negative control #1 mimic (p 0.15 and 0.11 for day 10 and 14 respectively, Fig. 6b).

Cyclin D1 is required for cell cycle G1/S transition [30]. To test for the corresponding functional effect of miR-511, we determined the cell cycle distribution of THP1 cells in miRNA mimic experiments via flow cytometry. Indeed, the percentage of cells in S-phase was significantly reduced in miR-511 mimic treatments on experimental days 6 and 9 (Fig. 6c).

### MiR-511 is associated with differentiation in THP1 cells

**Fig. 7:**
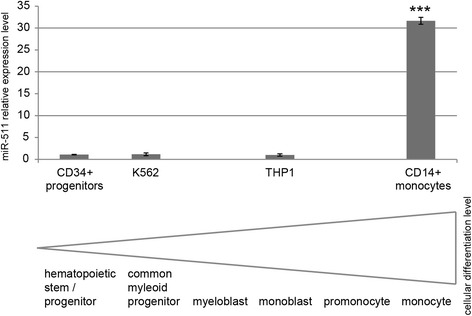
Mir-511 expression levels in primary hematopoietic stem / progenitor cells, leukemic cell lines and primary monocytes. MiR-511 expression level is shown in relation to the cellular differentiation level. *** *p* < 0.0005

Cyclin D1 has been described to inhibit ligand-induced PPAR-gamma function [31] while ligand activation of the PPAR-gamma/retinoid X receptor-alpha heterodimer in myelomonocytic cell lines is known to induce monocytic differentiation [32, 33]. Thus, expression of the miR-511 target cyclin D1 may indirectly influence monocytic differentiation. Additionally, miR-511 has been described to be upregulated after differentiating human blood monocytes into macrophages [29]. Concordantly, we observed miR-511 to be significantly upregulated (10.8-fold) 24 h after inducing differentiation of THP1 cells via 5 ng/ml phorbol 12-myristate 13-acetate (PMA) in three independent experiments (*p* < 0.05). In this setting, cellular differentiation was confirmed via cell adherence, morphological changes (cell protrusions, fusiform cells) and growth arrest. Additionally, although representing more differentiated cell types, we observed the leukemic cell lines THP1 and K562 to express similar low levels of miR-511 as primary CD34+ hematopoietic stem/progenitor cells, while primary CD14+ monocytes expressed significant higher miR-511 levels (Fig. 7).

In this context, we asked if miR-511 is able to promote cellular differentiation in the AML cell line THP1. For this purpose, we treated THP1 cells with miR-511 mimic or negative control mimics and analysed the expression of surface myeloid differentiation marker CD11b and CD38 [34] via flow cytometry. While CD11b was not expressed above background (isotype control) in any treatment (Additional file 2: Figure S4), CD38 signal to noise ratio was significantly higher in miR-511 treated compared to both negative control mimic treated THP1 cells and this effect intensified over the experimental time course (Fig. 6d). Compared to CD14+ monoblasts, untreated THP1 cells expressed 4.8-fold higher levels of CD38.

MiR-511 may thus exert pro-differentiation roles in THP1 cells, as in addition to being a marker for myeloid differentiation, CD38 has been described to promote cellular differentiation of HL-60 cells in response to 1,25-dihydroxyvitamin D3 and retinoic acid [34]. As CD38 is not a predicted target of miR-511 via TargetScan, this effect of miR-511 on monocytic differentiation is likely mediated via other target genes.

### MiR-511 augments migratory capability of THP1 cells

Migratory capability of myeloid cells is closely linked to their differentiation status: during normal monocyte maturation, cells gain the capability of locomotion [35]. In contrast to mature monocytes, AML monoblasts appear to lack the apparatus necessary for locomotion as neither chemotaxis nor random migration could be detected in this primary cell type [35].

We thus asked, if miR-511 expression is able to raise the migratory capability – as a sign of differentiation – of THP1 monoblasts. Indeed, we were able to observe that miR-511 mimic treatment significantly augments the capability of THP1 cells to migrate through 5 μm transwells (Fig. 6e).

## Discussion

We employed a quantitative profiling technique (LDAs) to analyze differential expression of miRNAs after efficient and specific MLL-AF9 depletion in THP1 cells and validated the results by single assay RT-qPCR for a subset of deregulated miRNAs. We excluded off-target effects by employing two distinct *MLL-AF9*-targeting as well as two distinct non-targeting control siRNA treatments and detected 21 miRNAs to be differentially expressed in *MLL-AF9* knockdown versus control treatments.

A causal explanation is still lacking for the majority of aberrantly expressed miRNAs in acute leukemia and aberrant expression of neighboring genes, genomic aberrations and specific regulatory factors can only explain the minority of dysregulated miRNAs [36]. Our study however, focused on the role of the epigenetic regulator MLL-AF9 alone, so that the observed differential regulation of miRNAs may be attributed to this factor.

MiR-196b has been previously described to be upregulated by MLL-AF9 employing Mll knockout or exogenous Mll / Mll-Af9 transduced murine cells [37–39]. We, however, observed a divergent expression of miR-196b levels between the two non-targeting control siRNA treatments which led to the exclusion of this miRNA from our differential miRNA expression signature. This observation suggests that siRNAs may have direct or indirect off-target effects not only on mRNA expression but also on miRNAs and corroborates the importance of using adequate controls for off-target effects in siRNA studies. The high stringency of our experimental design might thus explain the relatively low number of differentially expressed miRNAs we detected. Because miRNA processing is a specifically regulated process [12], it is not surprising that we did not detect miRNA host-genes to be differentially expressed in our data set.

Recently, in a number of *in vivo* studies, miRNAs have been found to be differentially expressed among distinct cytogenetic groups of AML. However, the specific signatures differed among studies, most likely due to the lack of standardized methods used by different groups [40]. This also includes the use of different miRNA profiling platforms and versions resulting in diverse coverages of quantified miRNAs. Nevertheless, some concordance was detected between our *in vitro* data set and previously published *in vivo* data. MiR-219 was described to be upregulated in AML with MLL-AF6 and MLL-AF9 versus other AML cases [41], while miR-589 was observed to be upregulated in MLL-rearranged AML versus normal controls [42].

To interpret possible functional and biological effects of the MLL-AF9 dependently expressed miRNAs, we employed *in silico* gene ontology analyses from 1) known direct as well as 2) predicted mRNA targets of these miRNAs. While the first analysis approach has the advantage to be based on *bona fide* MTIs instead of more or less reliable predictions, it is limited by the fact that only a small portion of actual MTIs have been experimentally validated up to date and thus this analysis can only yield a partial view on the actual roles the involved miRNAs play. The analysis approach employing predicted MTIs on the other hand is able to represent all miRNAs and a much larger proportion of target interactions, but will be hindered by false positive as well as false negative MTIs. To obtain the best possible comprehensive and reliable view on the functional roles these miRNAs play, we employed both analysis approaches.

We detected the known direct targets of our set of MLL-AF9 dependently expressed miRNAs to be mainly involved in proliferation and apoptosis, in functions related to monocyte / macrophage differentiation as well as in transcriptional regulation and cancer. Five of the miRNAs (miR-137, −214-3p, −301a-3p, −330-3p and −383-5p) emerged to possess target genes affecting all of these four categories, which may imply their relevance for leukemia. The gene ontology analysis of *in silico* predicted direct targets of MLL-AF9 dependently expressed miRNA implied cell cycle promoting and checkpoint decreasing roles as well as stem cell supporting and cellular stress response suppressive roles for these miRNAs.

To predict which biological functions may be predominantly influenced by MLL-AF9 via miRNA expression (as opposed to direct gene regulatory effects of MLL-AF9), we compared the gene ontology results from our previously published MLL-AF9 dependent gene expression profile [7] with the gene ontology results from known and predicted target genes of MLL-AF9 dependently expressed miRNAs. This analysis indicated that the cell cycle, Wnt signaling, adhesion, myeloid differentiation as well as the cellular response to stress may be preferentially influenced by MLL-AF9 via miRNA expression.

We observed enriched influence of MLL-AF9 on downstream transcriptional regulators both, via gene expression targeting as well as via miRNA expression targeting of MLL-AF9. However, due to the relative difficulty to therapeutically target transcription factors, e.g. via small molecule drugs, manipulation of these miRNAs may present a promising future opportunity to therapeutically target deregulated gene expression in MLL-AF9 positive leukemia.

Altogether, these gene ontology analyses show that the MLL-AF9 dependently expressed miRNAs are presumably involved in many leukemia relevant functions. Is has to be stressed however, that these analyses are not able to take into account the extent of MLL-AF9 dependent miRNA expression strength. Additionally, targeting of single gene transcripts by a miRNA is dependent on the cellular context and e.g. the present amount of transcripts from other direct target genes of that miRNA. Thus, while these analyses may suggest certain functional roles of the set of MLL-AF9 differentially regulated miRNAs in the context of leukemia, these functions will have to be experimentally validated.

To venture into this direction, we subsequently analyzed targets and functional roles of miR-511, which was one of the most deregulated miRNAs by MLL-AF9. To this end, we employed miRNA mimic mediated up-regulation of miR-511 in THP1 cells and subsequently analyzed a comprehensive gene expression profile as well as effects on functional level.

We were able to validate significant and strong up-regulatory effects of miR-511 on the expression of *CCL2*, *MMP9* and *IL1B* and significant down-regulatory effects on *PDGFA* and cyclin D1. Possible anti-leukemic roles of miR-511 via anti-angiogenesis, anti-proliferative as well as pro-differentiation and pro-immunosurveillance effects is suggested by known roles of these downstream genes in chemotactic activity [43], AML immunosurveillance [43], hematopoietic progenitor cell mobilization [44], macrophage migration [45], monocytic differentiation [46, 47] and tumor cell proliferation, angiogenesis and metastasis [48]. As such, miR-511 might be e.g. an alternative way to raise immunosurveillance against AML monoblasts via its effect to induce *CCL2* expression.

In addition to its classical role as cell cycle promoter, cyclin D1 has been reported to be involved in cancer drug resistance by being suppressed by a miRNA (let-7b), to be essential for migration in macrophages [49, 50] and might be indirectly involved in monocytic differentiation via inhibition of PPAR-gamma function [31], as the PPAR-gamma/retinoid X receptor-alpha heterodimer in myelomonocytic cell lines is known to induce monocytic differentiation [32]. Concordantly, we found miR-511 expression to be significantly higher in monocytes as compared to leukemic cell lines and hematopoietic stem/progenitor cells.

As miRNAs primarily act via post-transcriptional gene repression, the observed up-regulatory effects of miR-511 on *CCL2*, *MMP9* and *IL1B* may be indirect. Although this does not necessarily lessen their importance in miR-511 mediated cellular functions, we aimed at validating direct miR-511 targets. To this end we preformed dual luciferase assays with cloned full-length 3′-UTR sequences of *CCND1*, *FRFG3* and *PDGFA* and confirmed a significant repressive effect of miR-511 mimic on cyclin D1 3′UTR. We further succeeded to experimentally confirm miR-511 binding sites within the *CCND1* 3′UTR which validated cyclin D1 as a direct and specific target of miR-511. While cyclin D1 is not a direct target of *MLL* or *MLL* fusion genes such as *MLL-AF9* [51], this observation is in concordance with the likelihood of miR-511 targeting as predicted via TargetScan, which yielded a context + score of −0.43 for cyclin D1, while indicating a lower probability for *FGFR3* and *PDGFA* of being a miR-511 target (context + score −0.11 and not predicted, respectively).

Although the luciferase assays demonstrated cyclin D1 to be a direct target of miR-511, this experimental approach presents a relatively artificial system with overexpression of the target gene 3′UTR cloned into a luciferase vector and introduced into HEK cells. To address the regulation within the relevant cellular context, we additionally confirmed a suppressive effect of miR-511 on cyclin D1 protein expression in THP1 monoblasts via flow cytometry.

We further aimed to define cellular and functional consequences of miR-511 expression in the context of AML monoblasts. To this end, we were able to provide evidence for miR-511 to reduce the proportion of cells in S-phase of the cell cycle and to increase the migratory capability of the cells. Even though we detected THP1 cells to express 4.8-fold higher levels of CD38 compared to human primary CD14+ monocytes, CD38 is an early myeloid differentiation marker in myeloblasts [52] and, importantly, a known differentiation promoting ectoenzyme receptor [34]. Thus, miR-511 expression may promote monoblast differentiation as indicated by the raised expression of CD38.

As we did not observe significant effects on cellular proliferation within the experimental time frame (up to three days), these observations primarily suggest a role of miR-511 in monoblast differentiation, as cell cycle, cyclin D1 as well as migratory capability are also linked to myeloid differentiation: cell cycle lengthening is important for macrophage differentiation (e.g. via leading to an accumulation of the hematopoietic transcription factor PU.1) [53], decrease of cyclin D1 may induce monocytic differentiation via raised PPAR-gamma/retinoid X receptor-alpha function [31, 32] and migratory capability / locomotion is a trait gained during monoblastic differentiation [35]. Thus, besides a potential therapeutic use in leukemia vaccination approaches via its strong up-regulatory effect on *CCL2* expression which is known to raise immunosurveillance [43], miR-511 may prove useful in differentiation treatment strategies. The latter therapeutic approaches stimulate the leukemic cells to differentiate while often simultaneously inducing growth arrest and apoptosis [33]. Due to their usually low toxicity profile, these differentiation treatments may be especially useful in combination regimens with cytotoxic chemotherapies [33].

## Conclusions

We here present a *MLL-AF9* dependent miRNA expression profile encompassing 21 miRNAs and interpret the signature’s consequences via gene ontology analyses to influence a wide array of cellular functions important for leukemia maintenance. A comparison to functional roles of *MLL-AF9* dependent gene expression suggested that the cell cycle, Wnt signaling, the cellular response to stress, adhesion as well as myeloid differentiation may be preferentially influenced by *MLL-AF9* via miRNA expression. Furthermore, many transcriptional regulators are among the known and predicted miRNA target genes and thus therapeutic miRNA manipulation may present a possibility to target these hard-to-drug proteins in the future. We further present miR-511, which is strongly repressed by *MLL-AF9*, to upregulate *CCL2*, a chemokine ligand important for immunosurveillance, to directly target the key cell cycle regulator cyclin D1, to inhibit cell cycle progression, to increase cellular migration and to promote monoblastic differentiation. With these effects, miR-511 may have a potential therapeutic use as a pro-differentiation agent as well as in leukemia vaccination approaches.

## Additional files

Additional file 1:
**All (non-filtered) gene ontology results of known miRNAs targets.** DAVID gene ontology analysis was performed for all THP1-expressed, validated direct targets (extracted from miRTarBase) of *MLL-AF9* knockdown associated miRNAs. This file includes all extracted MTIs extracted from miRTarBase as well as all gene ontology results from DAVID analysis (clustered and non-clustered terms). (XLSX 125 kb) Additional file 2: Figure S1: Correlation between miRNA data from LDA and single assay RT-qPCR after *MLL-AF9* knockdown in THP1 cells. **Figure S2:** MiRNA-target gene associated functional annotation terms identified via DAVID (MiRTarBase targets). **Figure S3:** Venn diagram of miRNAs involved in relevant biological functions. **Figure S4:** Effect of miR-511 mimic on surface expression of monocytic marker CD11b. **Table S1:** RT-qPCR Primer information (DOC 673 kb) Additional file 3:
**All (non-filtered) gene ontology results of predicted miRNAs targets.** DAVID gene ontology analysis was performed for all THP1-expressed targets predicted via TargetScan with a minimum context + score of 0.4 of *MLL-AF9* knockdown associated miRNAs. This file includes all predicted MTIs as well as all gene ontology results from DAVID analysis (clustered and non-clustered terms) with a *p*-value below 0.1 and a fold enrichment above 1.5. (XLSX 475 kb) Additional file 4:
**Functional disease ontology (FunDO) analysis results of genes differentially regulated by miR-511 mimic treatment.** Analysis was used to extract leukemia and cancer related genes. (XLSX 22 kb)
