# Supplementary material for: The leukemogenic fusion gene MLL-AF9 alters microRNA expression pattern and inhibits monoblastic differentiation via miR-511 repression
Source: J Exp Clin Cancer Res. 2016 Jan 13;35:9. doi: 10.1186/s13046-016-0283-5 (PMC4712549; doi:10.1186/s13046-016-0283-5)
Supplement: Additional file 2: Figure S1: — Correlation between miRNA data from LDA and single assay RT-qPCR after MLL-AF9 knockdown in THP1 cells. Figure S2: MiRNA-target gene associated functional annotation terms identified via DAVID (MiRTarBase targets). Figure S3: Venn diagram of miRNAs involved in relevant biological functions. Figure S4: Effect of miR-511 mimic on surface expression of monocytic marker CD11b. Table S1: RT-qPCR Primer information (DOC 673 kb) [file 13046_2016_283_MOESM2_ESM.doc]

Additional file 2


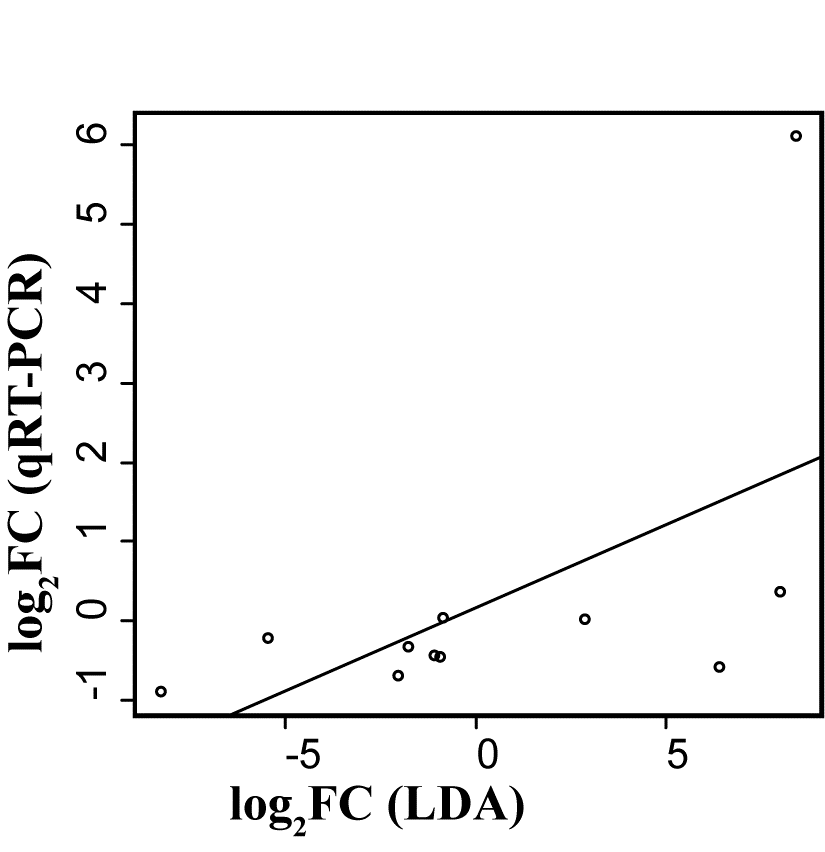


Additional file 2: Figure S1: **Correlation between miRNA data from LDA and single assay RT-qPCR after *MLL-AF9* knockdown in THP1 cells.** To test reliability of miRNA TaqMan low density array (LDA) data, single-assay RT-qPCR was performed for 11 miRNAs (which showed differential expression in LDA results) in additional sample pools of 2 independent experiments (significant correlation, Spearman’s *Rho* = 0.66, p=0.03). Linear regression (black line) and Spearman’s rank correlation coefficients including p values were calculated with R.


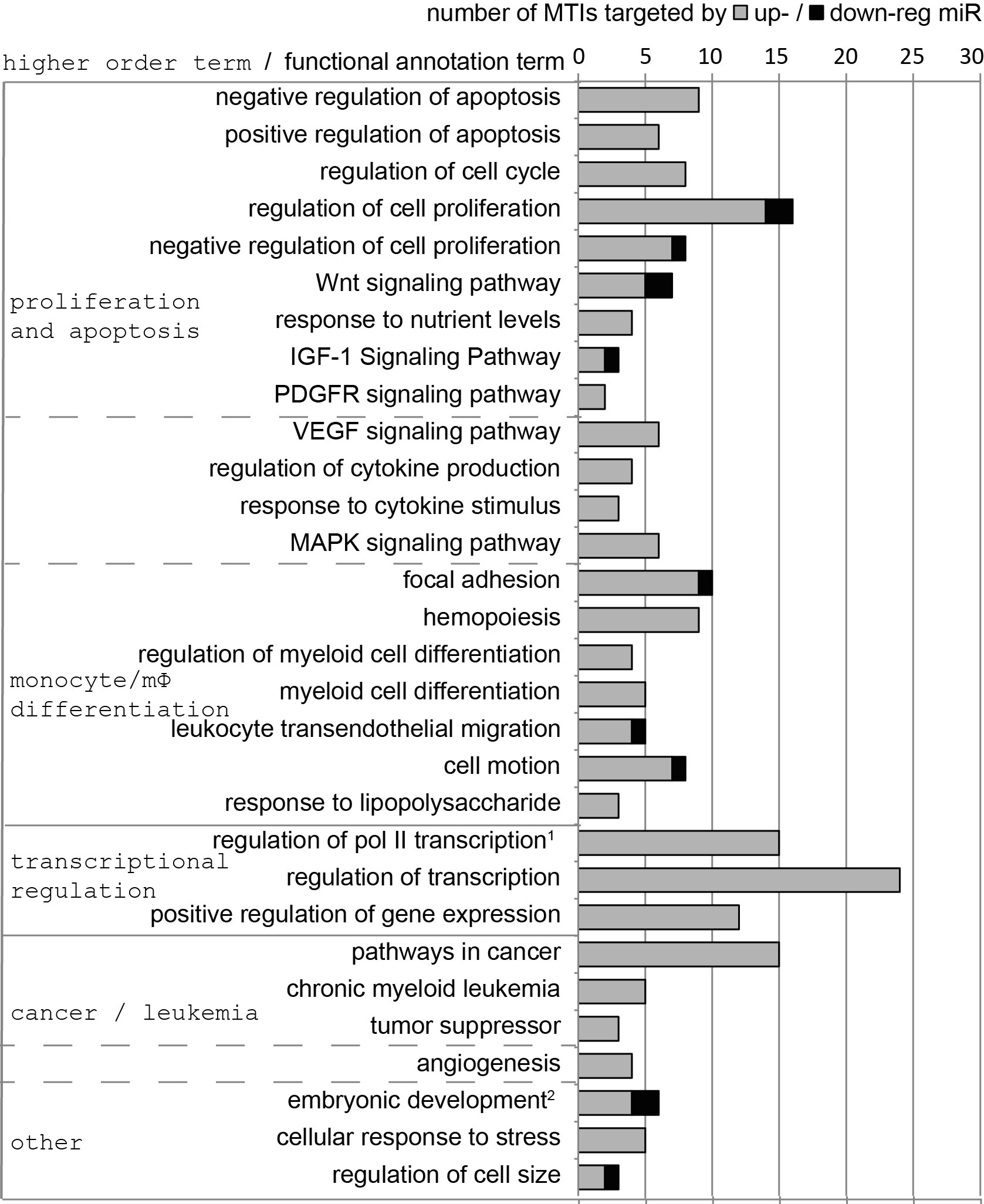


Additional file 2: Figure S2: **MiRNA-target gene associated functional annotation terms identified via DAVID.** Gene ontology analysis was performed for all THP1-expressed, validated direct targets (extracted from miRTarBase) of *MLL-AF9* knockdown associated miRNAs. Annotation terms were manually assorted to five higher-order terms according to the major role of the process in the biological setting under investigation. Annotation terms assigned to two higher-order terms are placed between these two and separated by dotted division lines. Some annotations were abbreviated as indicated by superscript numbers: ^1^regulation of transcription from RNA polymerase II promoter; ^2^embryonic development ending in birth or egg hatching; Φ macrophage. Columns show proportion of miRNA-target gene interactions (MTIs) of miRNAs which were up- (grey) or down-regulated (black) after MLL-AF9 depletion in THP1 cells and thus imply a target gene down- (grey) or up-regulation (black) in MLL-AF9 depleted cells. Most of the MTIs comprise up-regulated miRNAs, as 51 of the 56 MTIs from miRTarBase for our set of miRNAs concern MLL-AF9 dependently up-regulated miRNAs.


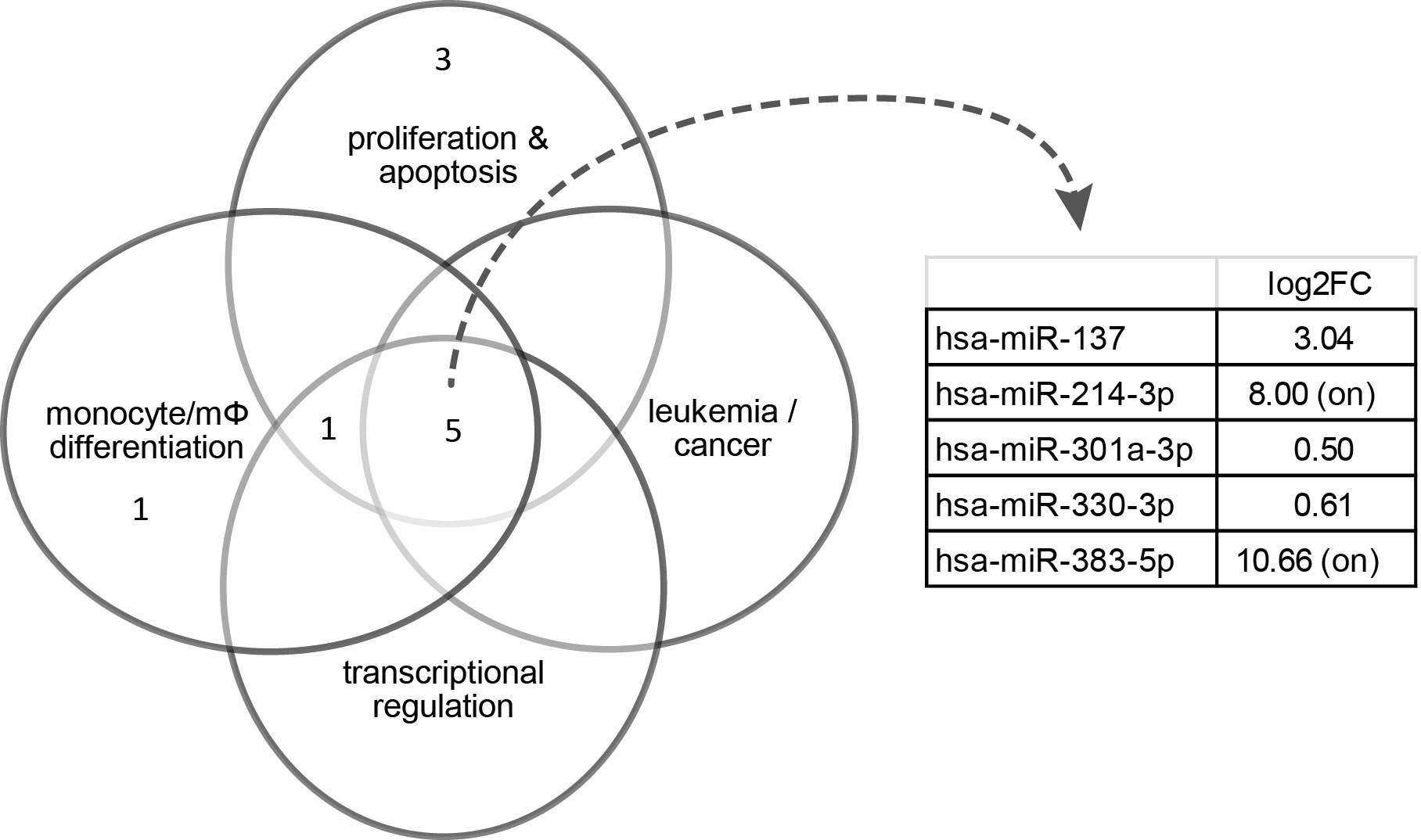


Additional file 2: Figure S3: **Venn diagram of miRNAs involved in relevant biological functions.** MiRNAs were assorted to higher-order functional annotations terms after DAVID analysis of validated miRNA targets from miRTarBase. MiRTarBase listed validated targets for 10 out of 21 MLL-AF9 dependently expressed miRNAs. Of these, five are associated with all four higher-order functional annotations terms as shown in this Venn diagram.


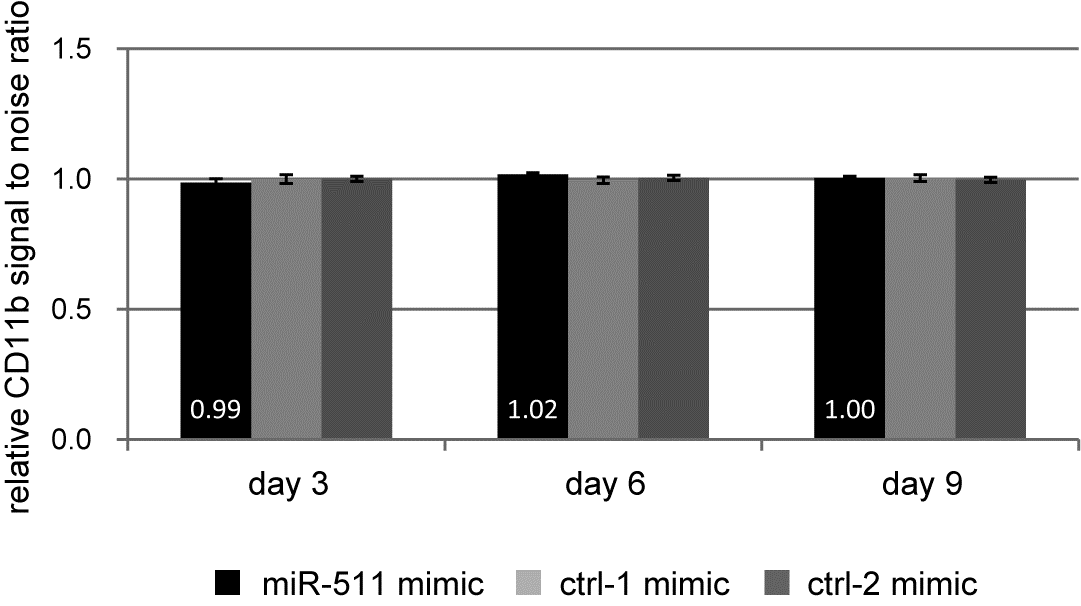


Additional file 2: Figure S4: **Effect of miR-511 mimic on surface expression of monocytic marker CD11b.** In neither treatment CD11b was expressed above isotype control.

Additional file 2: Table S1: RT-qPCR Primer information

| **Gene name** | **Primer name** | **Orientation** | **Sequence 5'-3'** | **Product size** |
| --- | --- | --- | --- | --- |
| CCL2 | CCL2 F | forward | GCTCATAGCAGCCACCTTCATTC | 289 |
| CCL2 | CCL2 R | reverse | CTTGGGTTGTGGAGTGAGTGTTC | 289 |
| CCND1 | CCND1-001 F | forward | CAAACACGCGCAGACCTTCG | 236 |
| CCND1 | CCND1-001 R | reverse | TTGACTCCAGCAGGGCTTCG | 236 |
| FGFR3 | FGFR3_v2 F | forward | CATCCCTGTGGAGGAGCTCTT | 183 |
| FGFR3 | FGFR3_v2 R | reverse | TCGGTGGACGTCACGGTAA | 183 |
| IL1B | IL1B F | forward | CTGTACCTGTCCTGCGTGTTG | 397 |
| IL1B | IL1B R | reverse | GGCAGTTGGGCATTGGTGTAG | 397 |
| KDM2B | KDM2B_2 F | forward | AGGGACTAAAGGATGCCCAGATG | 328 |
| KDM2B | KDM2B_2 R | reverse | CCACAGCGTTTGAAGAAGGACAG | 328 |
| MAPK11 | MAPK11_v2 F | forward | GATGAGAGCGTTGAGGCCAAG | 399 |
| MAPK11 | MAPK11_v2 R | reverse | TCCCAGTGGAGAGTGCAGTAG | 399 |
| MMP9 | MMP9 F | forward | TTCGACGTGAAGGCGCAGATG | 148 |
| MMP9 | MMP9 R | reverse | AACTCACGCGCCAGTAGAAGC | 148 |
| NEK8 | NEK8-001_v2 F | forward | GGGCATCAAGATGGCAATGGTAG | 204 |
| NEK8 | NEK8-001_v2 R | reverse | AGGGTGTTTCCATGGCAACAG | 204 |
| NSD1 | NSD1_v1 F | forward | CAGCTCGTCTCCTGCAAGAAAC | 186 |
| NSD1 | NSD1_v1 R | reverse | CATCCCTTCTCGATGCTGCTTAC | 186 |
| PARP1 | PARP1-003 F | forward | AGAAATGCAGCGAGAGCATCC | 361 |
| PARP1 | PARP1-003 R | reverse | AACATGGGCACCATACGCTTG | 361 |
| PDGFA | PDGFA-001_v3 F | forward | TCCGGATTATCGGGAAGAGGACACGGATG | 412 |
| PDGFA | PDGFA-001_v3 R | reverse | CCCGGACAGAAATCCAGTCTGCTGAGACC | 412 |
| PTP4A3 | PTP4A3_v4 F | forward | CGCTGGAGAAGGATGGCATCAC | 90 |
| PTP4A3 | PTP4A3_v4 R | reverse | GGCTCAGCCAGTCTTCCACTAC | 90 |
| RAPGEF3 | RAPGEF3 F | forward | ACCACTCAGAAGCCGAGTTTCC | 359 |
| RAPGEF3 | RAPGEF3 R | reverse | TCCTCCTTAGCTGCCAGTCATC | 359 |
| RPL13A | RPL13A F | forward | CCTGGAGGAGAAGAGGAAAGAGA | 126 |
| RPL13A | RPL13A R | reverse | TTGAGGACCTCTGTGTATTTGTCAA | 126 |
| SMURF2 | SMURF2_v1 F | forward | TGCACTAACAACCTGCCGAAAG | 356 |
| SMURF2 | SMURF2_v1 R | reverse | TGAATGTGGCCTCATGGCAAAG | 356 |
| SPAG6 | SPAG6_v1 F | forward | GCTGCCGCATGATAGCAAAGCTC | 201 |
| SPAG6 | SPAG6_v1 R | reverse | GGTTGATAGCTGTCCACCCTCTG | 201 |
| UBC | UBC F | forward | ATTTGGGTCGCGGTTCTTG | 133 |
| UBC | UBC R | reverse | TGCCTTGACATTCTCGATGGT | 133 |
| Unless indicated by citation, primers were designed utilizing Clone Manager Suite 7 (Sci-Ed Software, Cary, NC, USA). | | | | |

1. Vandesompele J, De Preter K, Pattyn F, Poppe B, Van Roy N, De Paepe A et al. Accurate normalization of real-time quantitative RT-PCR data by geometric averaging of multiple internal control genes. Genome Biology. 2002;3(7):research0034.1-research.12.
